# Optimal Sensor Placement Based on Eigenvalues Analysis for Sensing Deformation of Wing Frame Using iFEM

**DOI:** 10.3390/s18082424

**Published:** 2018-07-25

**Authors:** Yong Zhao, Jingli Du, Hong Bao, Qian Xu

**Affiliations:** 1 Key Laboratory of Electronic Equipment Structure Design of Ministry of Education, Xidian University, Xi’an 710071, China; henanzy1984@163.com (Y.Z.); jldu@mail.xidian.edu.cn (J.D.); 2 Xinjiang Observatory, National Astronomical Observatories, Chinese Academy of Sciences, Urumqi 830011, China; xuqian@xao.ac.cn

**Keywords:** deformation sensing, inverse Finite Element Method, Timoshenko beam theory, optimal placement of sensors, eigenvalue analysis

## Abstract

For real time monitoring of the wing state, in this paper, the inverse Finite Element Method (iFEM) is applied, which describes the displacement field of beam according to the Timoshenko theory, to sense the wing frame deformation. In order to maintain the accuracy and stability of frame deformation sensing with iFEM, an optimal placement model of strain sensors based on eigenvalue analysis is constructed. Through the model solution with the Particle Swarm Optimization (PSO) algorithm, two different optimal placement schemes of sensors are obtained. Finally, a simulation is performed on a simple cantilever beam and a static load experiment is conducted on an aluminum alloy wing frame. The results demonstrate that the iFEM is able to accurately sense the deformation of the wing frame, when the two optimal placement schemes of sensors are used.

## 1. Introduction

Deformation sensing in real-time is an essential technology for providing feedback to the actuation and control systems of smart structures, especially of next-generation aircrafts. Moreover, when the detailed state of structural deformation is known, other necessary response quantities, such as stress and failure criteria, can also be assessed [1,2,3]. A research of deformation sensing focuses on the utilization of in situ strain measurements, captured from a network of strain sensors, to estimate the structural deformation, which is commonly referred to as shape sensing. Specifically, fiber Bragg grating (FBG) sensors have been extensively studied for shape sensing due to the corresponding lightness, accuracy and ease of embedding [4,5,6,7].

Nevertheless, the structural deformation sensing from in situ strain data always represents an inverse problem. The inverse problem is frequently ill-posed, since it does not satisfy conditions of existence, uniqueness and stability [8,9]. Although many types of inverse problems and their applications have been proposed, few researchers have dealt with the deformation sensing of the wing shape. The current methods used to sense the wing shape can be divided into two categories [10]: one category focuses on the deformation sensing of the skin comprised of plate/shell structures to reflect the deformation of the wing shape and the other category emphasizes on the deformation sensing of a wing frame made of beam structures.

The effectiveness of the modal transformation scheme was analyzed and verified by Bogert et al. [11], in which the in-situ surface strain is used to sense the bending plate deformation. Despite the advantage of this method, accurate mode shapes and extensive eigenvalue analysis are required; for eigenvalue analysis, the elastic and inertial material properties need to be described in detail for application in high-fidelity finite element methods. On the basis of the least-squares and first-order plate theory, which includes the membrane and the bending and transverse shear deformations, a mathematical framework was developed by Tessler and Spangler to conduct the deformation sensing of plate and shell—called the inverse Finite Element Method (iFEM) [8,12]. The advantage of iFEM is that the structural deformations are sensed only from strain measurements, without the finite element model and a priori knowledge, such as damping, loading and material properties [13,14]. Through the iFEM framework and the Refined Zigzag Theory (RZT), Cerracchio and Gherlone et al. explored an effective way to sense the shape and stress of multilayered composites and sandwich structures [15,16,17]. Nevertheless, the kinematic variables in the above iFEM framework are derived from the three-node inverse-shell (i3-RZT) element or three-node inverse-plate (iMIN3) element, without including the hierarchical drilling rotation degrees-of-freedom (DOF). In order to extend the practical application of iFEM for the shape-sensing analysis of large-scale structures (such as the ship), a four-node quadrilateral inverse-shell element named iQS4 was formulated by Kefal et al. that utilized the kinematic assumptions of the first-order and transverse-shear deformation theory, to add the hierarchical drilling rotation DOF into the kinematic variables [18,19]. In a series of works [14,15,16,17,18,19,20,21,22,23], the capability of iFEM framework is verified through simulation or experimentation for 3D deformation sensing of plate/shell structures of aerospace vehicles and ships. However, for the aforementioned iFEM schemes, the strain sensors need to be attached on the top and bottom of the plate/shell structures. When the sensors are placed on the exterior surface of the structure, the protection mechanism are performed on the FBG or resistance strain sensors, which affects the aerodynamic performance of the wing. To avoid this problem, the strain sensors can be placed on the wing frame covered by the wing skin. If so, the wing frame deformation can be calculated to reflect the wing shape change.

In order to conduct the deformation sensing of beam structures, Ko et al. developed a load-independent scheme to approximate the beam curvature, named Ko’s Displacement Theory [24]. The proposed scheme employs Euler-Bernoulli beam theory to account for the beam deformation, in which the discrete surface strain measurements are integrated by using piece-wise continuous polynomials to sense the beam deformation. The experimental tests show that Ko’s Displacement Theory is sufficiently accurate for the deflection sensing of aircraft wings [25,26]. Regardless of its advantage, the scheme is only suitable for one-dimensional deformation of a beam structure, requiring a high number of strain sensors. According to Timoshenko beam theory, Gherlone et al. analyzed the displacement field of a constant cross-section beam, consequently constructing the relationship between the displacement and the surface strain data through iFEM [27,28]. Furthermore, Gherlone et al. conducted a comparative research on the aforementioned three shape sensing approaches: the iFEM, the modal transformation method and the Ko’s Displacement Theory [10]. It is found that the iFEM method is slightly more accurate and attractive than the other two methods for shape sensing. The experimental verifications demonstrate that the accuracy of iFEM for the beam deformation sensing is severely affected by the locations where the strain sensors are placed but the optimal placement criterion of the sensors is not mentioned [29]. Meanwhile, the stability and existence of iFEM are also severely affected by the locations where the strain sensors are placed. For instance, all sensors are placed parallel to the centroidal axes of the beam element.

The existing optimal placements of the strain sensors are based on the mode shape analysis of the structure, such as the modal kinetic energy (MKE) method, the effective independence (EI), the modal assurance criteria (MinMAC) and the drive point residue (DPR) [30,31,32,33]. Based on a mode approach, Geng et al. proposed an optimization scheme and improved genetic algorithms (GA) for optimal FBG sensor placement; consequently, the best reconstruction effects are obtained for the reconstruction of flexible plate structures [34]. Yang et al. developed a robust optimal sensor placement for uncertain structures [35]. In their schemes, the fitness function based on the Fisher information matrix (FIM) is derived and extended to the interval parameters. Genetic algorithm is employed to obtain the optimal solution. Being different to the traditional sensors, FBG sensors possess different detecting probe length at different angles. In view of the above difference, Yi et al. developed a detective model for FBG sensor which fit its feature based on probability model and the optimal result of the FBG sensor placement is solved by Particle Swarm Optimization (PSO) algorithm [36]. In Reference [37], Soman et al. proposed an optimal placement model based on Modal Identification (MI) and Accurate Mode Shape Expansion (AMSE) to place the strain sensors and accelerometers simultaneously. The results indicate that the use of a multi-type sensor system can improve the quality of Structure Health Monitoring (SHM). Given the fact that the iFEM reconstructs the structure deformation without the mode shape, all previous optimal placement schemes of the strain sensors are not suitable for iFEM.

When the strain sensors are placed on the ill-suited locations, the coefficient matrix of the equations may be ill-posed since the essence of deformation sensing with iFEM focuses on the solution of a linear system of equations. In [38,39,40], the optimal placement of sensors is regarded as the parameter identification of linear system of equations, using the condition number as the optimal object to construct the optimal model of sensors. When the condition number in a matrix is higher than 10^16^, the solution algorithm can return results with no accuracy at all; thus, such a matrix is numerically singular and linear systems with this matrix are not solved [39]. It is considered that a matrix is morbid when the condition number of the matrix is higher than 10^3^. Nevertheless, in our research, it is found that the condition number of the coefficient matrix in iFEM tends to fall into [10^3^, 10^16^] when the coefficient matrix is nonsingular; consequently, the bigger condition number in this range does not mean that the coefficient matrix is morbid. Therefore, the condition number is not suitable for the optimal sensor placement for iFEM.

This paper focuses on the construction of an optimal sensor placement model, based on eigenvalue analysis of the relationship between the sensor placement and the stability of sensing of the beam deformation with iFEM. This is conducted with an aim to maintain the sensing stability of iFEM. The contents of this paper are: firstly, the beam deformation sensing process through iFEM based on Timoshenko beam theory was reviewed. Following, the instability of sensing is discussed; consequently, the optimal placement model of strain sensors based on eigenvalue analysis is constructed. The two optimal results are obtained through the model solution with PSO algorithm. Moreover, a high-fidelity finite element model of the beam is constructed with ANSYS, which generate the discrete strain data to replace the experimental strain measurements captured from strain sensors and which produce the deformation data to replace the experimental deformation. This is executed to verify the robustness of iFEM under the optimal placement schemes. Finally, to examine the iFEM sensing capability for the two optimal placement schemes, deformation sensing test is conducted on an aluminum alloy wing frame model. 

## 2. Inverse Finite Element Method Algorithm for Beam Deformation

**Figure 1:**
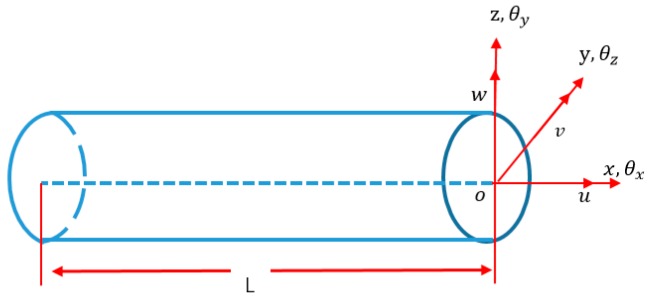
Beam kinematic variable fields.

In the iFEM algorithm, the displacement fields of an isotropic, straight and circular cross section beam element can be described from Timoshenko beam theory [27,28,29]:

(1) $$\{\begin{array}{c} u_{x}\left(x,y,z\right)=u\left(x\right)+z\theta_{y}\left(x\right)-y\theta_{z}\left(x\right)\\ v_{y}\left(x,y,z\right)=v\left(x\right)-z\theta_{x}\left(x\right)\\ u_{z}\left(x,y,z\right)=w\left(x\right)+y\theta_{x}\left(x\right) \end{array},$$

where, $u_{x},\;v_{y},w_{z}$ are the displacements along the x,y and z axes; u(x), v(x) and w(x) denote the displacements along the center axis (x∈[0,l], y=z=0); $\theta_{x}\left(x\right),\;\theta_{y}\left(x\right)\;and\;\theta_{z}\left(x\right)$ are the rotations around the three coordinate axes (see Figure 1). The six kinematic variables are grouped in vector form as $u\equiv {\left\{u\left(x\right),\;v\left(x\right),\;w\left(x\right),\;\theta_{x},\;\theta_{y},\;\theta_{z}\right\}}^{T}$.

According to the small-strain hypothesis, the linear strains are given by Equation (1):

(2) $$\{\begin{array}{c} \varepsilon_{x}\left(x,y,z\right)=e_{1}\left(x\right)+ze_{2}\left(x\right)+ye_{3}\left(x\right)\\ \gamma_{xz}\left(x,y\right)=e_{4}\left(x\right)+ye_{6}\left(x\right)\\ \gamma_{xy}\left(x,z\right)=e_{5}\left(x\right)-ze_{6}\left(x\right) \end{array},\;$$

where, the section strains $e\left(u\right)={\left[e_{1},\;e_{2},\ldots ,e_{6}\right]}^{T}$ are theoretically defined as:

(3) $$\;\begin{array}{ccc} e_{1}\left(x\right)=u_{x,x}\left(x\right) & e_{3}\left(x\right)=-\theta_{z,x}\left(x\right) & e_{5}\left(x\right)=v_{y,x}\left(x\right)-\theta_{z}\left(x\right)\\ e_{2}\left(x\right)=\theta_{y,x}\left(x\right) & e_{4}\left(x\right)=w_{z,x}\left(x\right)+\theta_{y}\left(x\right) & e_{6}\left(x\right)=\theta_{x,x}\left(x\right). \end{array}\;$$

The iFEM senses the beam deformation shape by minimizing a weighted least-squares function φ, containing the section strains vector $e^{\varepsilon }$ computed from in situ surface strain data and the theoretical section strains vector e(u) defined through Equation (3):

(4) $$\varphi \left(u\right)=\|e\left(u\right)-e^{\varepsilon }\|{}^{2}.$$

In the finite element framework, the kinematic variables vector ***u(x)*** is obtained through interpolation from certain shape functions N(x) and the nodal degrees of freedom $u^{e}$ as following:

(5) $$u\left(x\right)=N\left(x\right)u^{e}.$$

With regard to different loading cases, the orders of shape functions N(x) differ. For the end-node loads, the order of N(x) is $c^{0}$; whereas for the uniformly distributed loads, the order is $c^{1}$ [28]. Thus, the total least-square function is a sum of ***N*** individual element contributions $\varphi^{j}\left(u\left(x\right),e^{\varepsilon }\right)$:

(6) $$\;\varphi =\sum_{j=1}^{N}\varphi^{j}\left(u\left(x\right),e^{\varepsilon }\right).\;$$

Taking into account the effects of axial stretching, bending, twisting and transverse shearing, the element contributions $\varphi^{j}\left(u\left(x\right),e^{\varepsilon }\right)$ are given by the dot product as:

(7) $$\;\varphi^{j}\left(u\left(x\right),e^{\varepsilon }\right)=\sum_{k=1}^{6}w_{k}^{e}\varphi_{k}^{e},\;$$

with $\left\{w_{k}^{e}\right\}=\left\{w_{1}^{0},w_{2}^{0}\left(\frac{I_{y}^{e}}{A^{e}}\right),w_{3}^{0}\left(\frac{I_{z}^{e}}{A^{e}}\right),w_{4}^{0},w_{5}^{0},w_{6}^{0}\left(\frac{I_{p}^{e}}{A^{e}}\right)\right\}$

(8) $$\;\varphi_{k}^{e}=\frac{l}{n}\overset{n}{\underset{i=1}{\sum }}{\left[e_{k}\left(x_{i}\right)-e_{k}^{\varepsilon }\left(x_{i}\right)\right]}^{2}\left(k=1,2,\ldots ,6\right),\;$$

where, $w_{k}^{0}\left(k=1,2,\ldots ,6\right)$ are identically set as 1; $A^{e}$, $I_{y}^{e}$, $I_{z}^{e}$ and $I_{p}^{e}$ are the cross-section area, the second moments of the area according to the y- and z-axis and the polar moment of the area of the beam element, respectively. l is the length of the beam element; $x_{i}\left(0\leq x_{i}\leq l\right)$ and *n* are the axial coordinate of the locations where the section strains are evaluated and the number of locations, respectively. For the end-node loads, $n_{min}=2$ and for the uniformly distributed loads, $n_{min}=3$ [27,28,29,41]. 

The invoking of Equations (3) and (5) results in the theoretical section strains as following:

(9) $$\;e\left(x\right)=B\left(x\right)u^{e},\;$$

where, the matrix B(x) contains the derivatives of the shape functions N(x). 

Substituting Equation (9) into Equation (8), Equation (4) yields the following quadratic form:

(10) $$\;\varphi \left(u\right)=\frac{1}{2}{\left(u^{e}\right)}^{T}k^{e}u^{e}-{\left(u^{e}\right)}^{T}f^{e}+C^{e},\;$$

where, $C^{e}$ is a constant vector; $k^{e}$ and $f^{e}$ are defined as follows:

(11) $$\;k_{k}^{e}=\frac{L}{n}\overset{n}{\underset{i=1}{\sum }}\left[B_{k}^{T}\left(x_{i}\right)B_{k}\left(x_{i}\right)\right]\;\;\;\;f_{k}^{e}=\frac{L}{n}\overset{n}{\underset{i=1}{\sum }}\left[B_{k}^{T}\left(x_{i}\right)e_{k}^{\varepsilon }\left(x_{i}\right)\right].$$

Finally, the minimization of function φ(u) in Equation (10) in terms of $u^{e}$ yields the sensing equation for the beam element deformation:

(12) $$\;k^{e}u^{e}=f^{e}.$$

**Figure 2:**
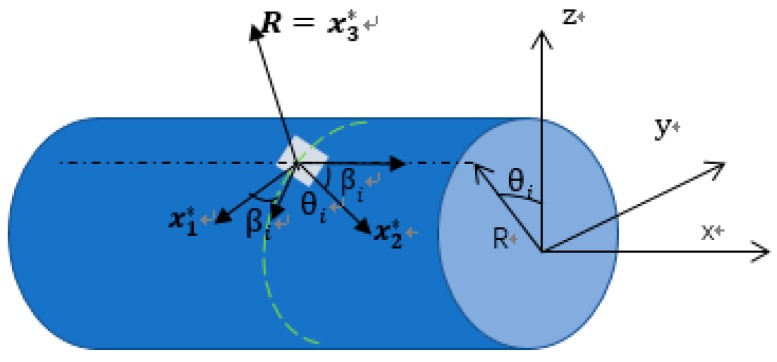
Location and coordinate of strain sensor placed on the external beam surface.

The two key steps in iFEM are: (1) the selection of suitable shape functions for the beam deformation sensing with iFEM; (2) the calculation of section strains from the measured surface strain data. For step (1), literatures [27,28,29,41,42] provide the detailed derivation process and results. For step (2), the section strains are computed through the following equation [27,28]:

$$\;\varepsilon \left(x_{i},\theta_{i},\beta_{i}\right)=e_{1}^{\varepsilon }\left(x_{i}\right)\left(c_{\beta }^{2}-vs_{\beta }^{2}\right)+e_{2}^{\varepsilon }\left(x_{i}\right)\left(c_{\beta }^{2}-vs_{\beta }^{2}\right)s_{\theta }R+e_{3}^{\varepsilon }\left(x_{i}\right)\left(c_{\beta }^{2}-vs_{\beta }^{2}\right)c_{\theta }R\;$$

$$+e_{4}^{\varepsilon }\left(x_{i}\right)c_{\beta }s_{\beta }c_{\theta }-e_{5}^{\varepsilon }\left(x_{i}\right)c_{\beta }s_{\beta }s_{\theta }+e_{6}^{\varepsilon }\left(x_{i}\right)c_{\beta }s_{\beta }R\;\;\;\;=\left[c_{\beta }^{2}-vs_{\beta }^{2},\left(c_{\beta }^{2}-vs_{\beta }^{2}\right)s_{\theta }R,\left(c_{\beta }^{2}-vs_{\beta }^{2}\right)c_{\theta }R,c_{\beta }s_{\beta }c_{\theta },c_{\beta }s_{\beta }s_{\theta },c_{\beta }s_{\beta }R\right]\times e^{\varepsilon }\left(x_{i}\right)$$

$$\;=T\left(x_{i},\theta_{i},\beta_{i}\right)\times e^{\varepsilon }\left(x_{i}\right)\;$$

(13) $$\mathrm{with}\;c_{\beta }\;\equiv \cos\beta_{i},s_{\beta }\equiv sin\beta_{i},c_{\theta }\equiv cos\theta_{i},s_{\theta }\equiv sin\theta_{i},$$

where, $e^{\varepsilon }\left(x_{i}\right)=\{e_{1}^{\varepsilon }\left(x_{i}\right),e_{2}^{\varepsilon }\left(x_{i}\right),\ldots ,e_{6}^{\varepsilon }\left(x_{i}\right)|\left(i=1,2,\ldots ,m\right)\}$ is the in-situ section strains vector at location $x_{i}$, along the *x*-axis. *R* is the external radius of the beam element. $\varepsilon \left(x_{i},\theta_{i},\beta_{i}\right)$ denotes the measured surface strain at the location $\left(x_{i},\theta_{i},\beta_{i}\right)$, which is signed by the cylindrical coordinate system (see Figure 2). $T\left(x_{i},\theta_{i},\beta_{i}\right)$ indicates the transformation relationship between the surface strain measurements and the section strains.

## 3. Construction of Optimal Placement of Sensors

**Figure 3:**
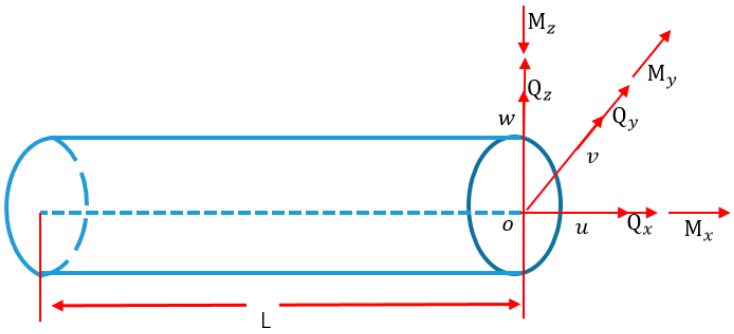
Beam section forces and moments.

When the section strains vector $e^{\varepsilon }$ is calculated from the surface strain measurements only with Equation (13), the strain measurements $\varepsilon \left(x_{i},\;\theta_{i},\;\beta_{i}\right)$ (*i* = 1, 2…6) and the section strains vector $e^{\varepsilon }$ must be distributed in one cross-section. Consequently, 6×n strain measurements are required to calculate the n section strain vectors. In the case of end-node loads, 12 strain measurements are required for the calculation of Section 2 strain vectors; in the case of uniformly distributed loads, 18 strain measurements are required for the calculation of Section 3 strain vectors. As aforementioned in the literatures [27,28,29,41], the number of strain measurements is reduced through the constitutive equations of Equation (14) (refer to Figure 3) and the equilibrium equations of Equation (15).

(14) $$\begin{array}{lll} N=A_{x}e_{1} & Q_{y}=G_{y}e_{5} & Q_{z}=G_{z}e_{4}\\ M_{x}=J_{x}e_{6} & M_{y}=D_{y}e_{2} & M_{z}=D_{z}e_{3} \end{array},$$

where, the section forces ($N,\;Q_{y}\;and\;Q_{z}$) and moments ($M_{x},\;M_{y}\;and\;M_{z}$) are related to the section strains $e_{i}\left(x\right)$. $A_{x}=EA$ is the axial rigidity, where A is the area of the cross-section of the beam element. $G_{y}=k_{y}^{2}GA$, $G_{z}=k_{z}^{2}GA$ are the shear rigidities with $k_{y}^{2}$ and $k_{z}^{2}$ denoting the shear correction factors, where *G* is the shear modulus. For the thin-walled section, $k_{y}^{2}$ = $k_{z}^{2}$ = 0.531; for the thick-walled section, $k_{y}^{2}$ = $k_{z}^{2}$ = 0.62; and for the solid section, $k_{y}^{2}$ = $k_{z}^{2}$ = 0.887 [28]. $J_{x}=GI_{p}$ is the torsional rigidity. $D_{y}=EI_{y}$ and $D_{z}=EI_{z}$ denote the bending rigidities.

(15) $$\begin{array}{ccc} \frac{\partial N}{\partial x}+q_{x}=0 & \frac{\partial Q_{y}}{\partial x}+q_{y}=0 & \frac{\partial Q_{z}}{\partial x}+q_{z}=0\\ \frac{\partial M_{x}}{\partial x}=0 & \frac{\partial M_{y}}{\partial x}-Q_{z}=0 & \frac{\partial M_{z}}{\partial x}-Q_{y}=0. \end{array}\;$$

When the forms of distribution loads $q_{x}$, $q_{y}$ and $q_{z}$ are known, the forms of the section strains $e^{\varepsilon }$ can be estimated. For the end-node loads, the section stains $e_{1}^{\varepsilon }\left(x_{i}\right)$, $e_{4}^{\varepsilon }\left(x_{i}\right)$, $e_{5}^{\varepsilon }\left(x_{i}\right)$ and $e_{6}^{\varepsilon }\left(x_{i}\right)$are constant and $\;e_{2}^{\varepsilon }\left(x_{i}\right)\;\mathrm{and}\;e_{3}^{\varepsilon }\left(x_{i}\right)$ are linear. For the uniformly distributed transverse loads, the section strains $e_{1}^{\varepsilon }\left(x_{i}\right)\;\mathrm{and}\;e_{6}^{\varepsilon }\left(x_{i}\right)$ are constant, $e_{4}^{\varepsilon }\left(x_{i}\right)\;\mathrm{and}\;e_{5}^{\varepsilon }\left(x_{i}\right)$ are linear and $e_{2}^{\varepsilon }\left(x_{i}\right)\;\mathrm{and}\;e_{3}^{\varepsilon }\left(x_{i}\right)$ are parabolic. $e_{4}^{\varepsilon }\left(x_{i}\right)=\frac{EI_{z}}{Gk_{y}^{2}A}e_{2,\;x}^{\varepsilon }\left(x_{i}\right)=m_{1}e_{2,x}^{\varepsilon }\left(x_{i}\right),e_{5}^{\varepsilon }\left(x_{i}\right)=\frac{EI_{y}}{Gk_{z}^{2}A}e_{3,x}^{\varepsilon }\left(x_{i}\right)=m_{2}e_{3,x}^{\varepsilon }\left(x_{i}\right)\;$[28,41]. Through the aforementioned results, the section strains are calculated as:

For the end-node loads, the section strains are expressed as:

(16) $$\begin{array}{ccc} e_{1}^{\varepsilon }\left(x_{i}\right)=a_{1} & e_{2}^{\varepsilon }\left(x_{i}\right)=a_{2}x_{i}+a_{4} & e_{4}^{\varepsilon }\left(x_{i}\right)=m_{1}a_{2}\\ e_{6}^{\varepsilon }\left(x_{i}\right)=a_{6} & e_{3}^{\varepsilon }\left(x_{i}\right)=a_{3}x_{i}+a_{5} & e_{5}^{\varepsilon }\left(x_{i}\right)=m_{2}a_{3} \end{array},$$

or in a matrix equation:

(17) $$e^{\varepsilon }\left(x_{i}\right)={\left\{e_{1}^{\varepsilon }\left(x_{i}\right),e_{2}^{\varepsilon }\left(x_{i}\right),\ldots ,e_{6}^{\varepsilon }\left(x_{i}\right)\right\}}^{T}\;\;\;\;=\left[\begin{array}{c} \begin{array}{c} 1\\ 0\\ 0 \end{array}\;\begin{array}{c} 0\\ x_{i}\\ 0 \end{array}\;\begin{array}{c} 0\\ 0\\ x_{i} \end{array}\;\\ \begin{array}{c} 0\\ 0\\ 0 \end{array}\;\begin{array}{c} m_{1}\\ 0\\ 0 \end{array}\;\begin{array}{c} 0\\ m_{2}\\ 0 \end{array} \end{array}\begin{array}{c} \begin{array}{c} 0\\ 1\\ 0 \end{array}\;\begin{array}{c} 0\\ 0\\ 1 \end{array}\;\begin{array}{c} 0\\ 0\\ 0 \end{array}\;\\ \begin{array}{c} 0\\ 0\\ 0 \end{array}\;\begin{array}{c} 0\\ 0\\ 0 \end{array}\;\begin{array}{c} 0\\ 0\\ 1 \end{array} \end{array}\right]\times {\left[a_{1},a_{2},a_{3},a_{4},a_{5},a_{6}\right]}^{T}=T1\left(x_{i}\right)\times p_{1},$$

where, $p_{1}={\left[a_{1},a_{2},a_{3},a_{4},a_{5},a_{6}\right]}^{T}$ is a constant parameters vector; $T1_{x_{i}}$ indicates the transfer matrix between $p_{1}$ and the section strains vector $e^{\varepsilon }\left(x_{i}\right)$.

For the uniformly distributed transverse loads, the section strains are expressed as:

(18) $$\begin{array}{ccc} e_{1}^{\varepsilon }\left(x_{i}\right)=b_{1} & e_{2}^{\varepsilon }\left(x_{i}\right)=b_{2}x_{i}^{2}+b_{3}x_{i}+b_{4} & e_{4}^{\varepsilon }\left(x_{i}\right)=2m_{1}b_{2}x_{i}+m_{1}b_{3}\\ e_{6}^{\varepsilon }\left(x_{i}\right)=b_{8} & e_{3}^{\varepsilon }\left(x_{i}\right)=b_{5}x_{i}^{2}+b_{6}x_{i}+b_{7} & e_{5}^{\varepsilon }\left(x_{i}\right)=2m_{2}b_{5}x_{i}+m_{2}b_{6} \end{array},$$

or in a matrix equation:

$$e^{\varepsilon }\left(x_{i}\right)={\left\{e_{1}^{\varepsilon }\left(x_{i}\right),e_{2}^{\varepsilon }\left(x_{i}\right),\ldots ,e_{6}^{\varepsilon }\left(x_{i}\right)\right\}}^{T}\;$$

(19) $$=\left[\begin{array}{cccccccc} 1 & 0 & 0 & 0 & 0 & 0 & 0 & 0\\ 0 & x_{i}^{2} & x_{i} & 1 & 0 & 0 & 0 & 0\\ 0 & 0 & 0 & 0 & x_{i}^{2} & x_{i} & 1 & 0\\ 0 & 2m_{1}x_{i} & m_{1} & 0 & 0 & 0 & 0 & 0\\ 0 & 0 & 0 & 0 & 2m_{2}x_{i} & m_{2} & 0 & 0\\ 0 & 0 & 0 & 0 & 0 & 0 & 0 & 1 \end{array}\right]\times {\left[b_{1},b_{2},b_{3},b_{4},b_{5},b_{6},b_{7},b_{8}\right]}^{T}$$

$$=T2\left(x_{i}\right)\times p_{2},$$

where, $p_{2}={\left[b_{1},b_{2},b_{3},b_{4},b_{5},b_{6},b_{7},b_{8}\right]}^{T}$ is a constant parameters vector; $T2_{x_{i}}$ indicates the transfer matrix between p2 and the section strains vector $e^{\varepsilon }\left(x_{i}\right)$. Following, the relationship between the undetermined parameters vector $p_{1}\;or\;p_{2}$ and any measured strain data $\varepsilon \left(x_{i},\;\theta_{i},\beta_{i}\right)$ is expressed as:

(20) $$\;\varepsilon \left(x_{i},\theta_{i},\beta_{i}\right)=T\left(x_{i},\theta_{i},\beta_{i}\right)T1\left(x_{i}\right)\times \;\varepsilon \left(x_{i},\theta_{i},\beta_{i}\right)=T\left(x_{i},\theta_{i},\beta_{i}\right)T2\left(x_{i}\right)\times p_{2}.$$

When the parameters vector p1 or p2 is solved, the arbitrary section strains vector is calculated through Equations (16) and (18).

(21) $$\;e^{\varepsilon }\left(x_{j}\right)=T1\left(x_{j}\right)\times {\left(T\left(x_{i},\theta_{i},\beta_{i}\right)T1\left(x_{i}\right)\right)}^{-1}\times \varepsilon \left(x_{i},\theta_{i},\beta_{i}\right)\;\mathrm{or}\;e^{\varepsilon }\left(x_{j}\right)=T2\left(x_{j}\right)\times {\left(T\left(x_{i},\theta_{i},\beta_{i}\right)T2\left(x_{i}\right)\right)}^{-1}\times \varepsilon (x_{i},\theta_{i},\beta_{i}),$$

where, i=1, 2,…n is the location where the section strains vector is calculated; n is the number of sections stated in Equation (8); j=1, 2,…,m and m is the minimum number of the strain sensors used to capture the surface strains. The value of m is different under different loading cases analyzed through Equations (16) and (18). For the end-node loads, m=6, whereas for the uniformly distributed loads, m=8 [28]. When the section strains vector $e^{\varepsilon }$ is obtained, the kinematic variables $u^{e}$ can be directly calculated from Equation (12).

However, through in-depth research, it is found that the transformation matrices $T\left(x_{i},\;\theta_{i},\;\beta_{i}\right)T1\left(x_{i}\right)$ and $T\left(x_{i},\;\theta_{i},\beta_{i}\right)T2\left(x_{i}\right)$ are ill-conditioned or even singular, when the strain sensors are placed at the inappropriate locations along the beam surface, such as $\beta_{i}$ in Equation (13) set to the same value $0^{o}$. Thus, the deformation reconstructed from Equation (12) does not necessarily satisfy the conditions of existence, uniqueness and stability [8]. 

**Figure 4:**
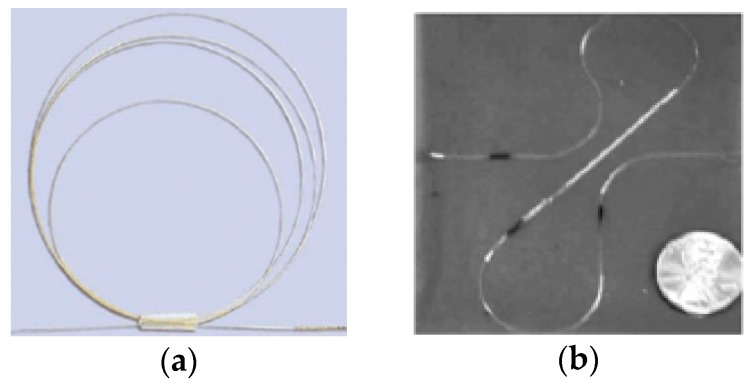
Fiber Bragg grating (FBG) sensor (**a**) and FBG strain rosette (**b**).

The constant parameter vectors p1 and p2 determination is regarded as the solution of a linear system of equations. The stability of Equation (20) solution depends on whether the product matrices $T\left(x_{i},\theta_{i},\;\beta_{i}\right)T1\left(x_{i}\right)$ and $T\left(x_{i},\;\theta_{i},\;\beta_{i}\right)T2\left(x_{i}\right)$ are well-conditioned or ill-conditioned. In actual measurements, the sensor placement is different from the pre-set placement. This means that one sensor is set at the node $\left(x_{i},\;\theta_{i},\;\beta_{i}\right)$ ideally but the actual sensor location might be at the node $\left(x_{i}^{\prime },\;\theta_{i}^{\prime },\;\beta_{i}^{\prime }\right)$ and the strain measurement contains the noise. Therefore, the strain input errors are added to Equation (20):

(22) $$\;\;\varepsilon \left(x_{i},\;\theta_{i},\beta_{i}\right)+\Delta \varepsilon \left(x_{i},\theta_{i},\beta_{i}\right)=T\left(x_{i},\theta_{i},\beta_{i}\right)\times H\left(x_{i}\right)\times \left(p+\Delta p\right)\;\Delta \varepsilon \left(x_{i},\theta_{i},\beta_{i}\right)=\varepsilon \left(x_{i}^{\prime },\theta_{i}^{\prime },\beta_{i}^{\prime }\right)-\varepsilon \left(x_{i},\theta_{i},\beta_{i}\right)\;(i=1,\;2,\ldots ,m),$$

where, $\Delta \varepsilon \left(x_{i},\theta_{i},\beta_{i}\right)$ indicate the strain input errors, which couple the errors of sensor placement with the noise of the strain measurement system. $\varepsilon \left(x_{i},\theta_{i},\beta_{i}\right)$ and $\varepsilon \left(x_{i}^{\prime },\theta_{i}^{\prime },\beta_{i}^{\prime }\right)$ are the ideal strain input at the node $\left(x_{i},\theta_{i},\beta_{i}\right)$ and the actual strain input, respectively. H=T1 or T2, Δp is the errors vector, resulting from the above errors. Following, the relative error between Δp and p is estimated as:

(23) $$\frac{\left\|\Delta p\right\|}{\left\|p\right\|}\leq \|A\|\times \|A^{-1}\|\frac{\left\|\Delta \varepsilon \right\|}{\left\|\varepsilon \right\|}\;=cond\left(A\right)\frac{\left\|\Delta \varepsilon \right\|}{\left\|\varepsilon \right\|}\;with\;A=T\left(x_{i},\theta_{i},\beta_{i}\right)H\left(x_{i}\right),\;cond\left(A\right)=\|A\|\times \|A^{-1}\|,$$

where, ‖·‖ indicates the matrix norm and cond(A) signifies the condition number of matrix A. To a certain extent, the condition number can reflect the morbidity degree of a matrix. From Equation (23), it is observed that the relative error is high and the matrix is ill-posed, when the condition number is high. Kunsoo Huh and J.L. Stein proposed that the reason for the morbidity of a matrix is that a good distribution of eigenvalues for the matrix does not exist. For well distributed eigenvalues, large differences among the matrix eigenvalues exist [43], such as the high value in $min\left|\lambda_{j}-\lambda_{k}\right|(j\neq k$, $\lambda_{j}$ and $\lambda_{k}$ are the eigenvalues of the matrix). Therefore, the optimal placement model of sensors is constructed as:

(24) $$find\;f\left(A_{\left(x,\theta ,\beta \right)}\right)=max\left(\min\left|\lambda_{j}-\lambda_{k}\right|\right)\left(j\neq k,\;\;j,\;\;k=1,\;2,\ldots ,m\right),\;\;\left(\left(x,\theta ,\beta \right)=\left[x_{i},\theta_{i},\beta_{i},\ldots ,x_{m},\theta_{m},\beta_{m}\right]\right)\;s.t.\;x_{i}\in \left[L/5,\;\;4L/5\right],\;\theta_{i}\epsilon \left[-180^{{}^{\circ}},180^{{}^{\circ}}\right]\;\;\beta_{i}=0^{{}^{\circ}}\;or\;45^{{}^{\circ}},\;i=1,\;2\ldots ,m$$

where, $\lambda_{i}$(*i* = 1, 2,…,*m*) are the eigenvalues of the matrix A. (x, θ, β) represents the locations where the sensors are placed and m is the number of the sensors used to capture the strain data of the structure. For the end-node loads, m=6, whereas for the uniformly distributed loads, m=8 [28]. In view of application environment in engineering, it is difficult to set FBG strain sensors on the clamped node and on the free end node of the beam element. Moreover, the curve of the beam surface affects the strain measurement accuracy when a non-zero angle exists between the sensor and the generatrix of the beam surface [29]. Thus, it is difficult to set two or more FBG sensors at one node along the surface of the beam element, as the fiber sensors are not stacked (see Figure 4). Consequently, $x_{i}\in \left[L/5,\;4L/5\right]$ is set, where only one sensor is placed at $\beta_{i}=45^{{}^{\circ}}$ and the other sensors are placed at $\beta_{i}=0^{{}^{\circ}}$. 

The optimal placement model of Equation (24) is a multi-parameter optimization problem, which is solved by using the PSO algorithm with good convergence speed. The optimal result is efficiently obtained in hyperspace. 

In a population that includes *N* particles, the status of the *i-th* particle is described by the current location and the current velocity. $a_{i}={\left(x_{i1},\theta_{i1},\beta_{i1},\ldots ,x_{im,}\theta_{im},\beta_{im}\right)}_{1*3m}$ is the current location of the *i-th* particle and $v_{i}={\left(v_{x1},v_{\theta 1},v_{\beta 1}\ldots ,v_{xm},v_{\theta m},v_{\beta m}\right)}_{1*3m}$ is the current velocity of the *i-th* particle (i=1, 2,…, N;m is the number of strain sensors). The fitness function of PSO algorithm is defined as:

$$f(a_{i})=f\left(A_{\left(x,\theta ,\beta \right)}\right)=max\left(\min\left|\lambda_{j}-\lambda_{k}\right|\right)\left(j\neq k,j,k=1,2,\ldots ,m\right),$$

(25) $$(\left(x,\theta ,\beta \right)=\left(x_{i1},\theta_{i1},\beta_{i1},\ldots ,x_{im,}\theta_{im},\beta_{im}{)}_{1*3m}\right),$$

and the updated velocity and location are expressed as:

(26) $$\;v_{i}^{k+1}=\alpha v_{i}^{k}+c_{1}r_{1}\times \left(p_{i}^{k}-a_{i}^{k}\right)+c_{2}r_{2}\times \left(p_{g}^{k}-a_{i}^{k}\right)\;a_{i}^{k+1}=a_{i}^{k}+v_{i}^{k+1},$$

where, $\alpha ,\;c_{1}\;and\;c_{2}$ are constants; $r_{1}\;and\;r_{2}$ are uniformly distributed random numbers between 0 and 1; $a_{i}^{k}$ and $v_{i}^{k}$ arethe current location and the current velocity of the *i-th* particle in the *k-th* iteration, respectively; $p_{i}^{k}$ is the individual best location of the *i-th* particle at step *k*; $p_{g}^{k}$ is the local best location of the population at step *k*. $p_{i}^{k}$ and $p_{g}^{k}$ are determined as:

(27) $$\;p_{i}^{k}=\{\begin{array}{c} p_{i}^{k-1},\;f\left(a_{i}^{k}\right)\leq f\left(p_{i}^{k}\right)\\ a_{i}^{k},\;f\left(a_{i}^{k}\right)>f\left(p_{i}^{k}\right) \end{array}\;\;p_{g}^{k}\in \left\{p_{0}^{k},p_{1}^{k},\ldots ,p_{N}^{k}\right\}|g_{loc}^{k}=f\left(p_{g}^{k}\right)=\max\left\{f\left(p_{0}^{k}\right),f\left(p_{1}^{k}\right),\ldots ,f\left(p_{N}^{k}\right)\right\}\;g_{glo}^{k}=max\left\{g_{loc}^{0},g_{loc}^{1},\ldots ,g_{loc}^{k}\right\},$$

where, $g_{loc}^{k}$ and $g_{glo}^{k}$ are defined as the local extremum and the global extremum of the population, respectively. The key steps of the optimal model Equation (24) solution with PSO algorithm are described as:

Step 1. PSO algorithm initializing. The size of the particle swarm is set to *N* = 50 and the maximum iteration is set to $k_{max}=1000$; the initial location $a_{0}$ and the initial velocity $v_{0}$ are set to the random values included in the constraint of Equation (24). The corresponding fitness value of each particle, which is calculated from the fitness function Equation (25), is set as the initial individual extremum $f\left(p_{i}^{0}\right)$, (*i* = 1,2,…,*N*). Following, the initial local extremum $g_{loc}^{0}$ and the global extremum $g_{best}^{0}$ of the population at the initial phase are selected for Equation (27). The threshold of the algorithm breaking is set to M=1000. When the global extremum $g_{best}^{0}$ is equal to M, the algorithm breaks and the current particle location is the optimal placement of the sensors. Else Step 2 will follow.
Step 2. Velocity and location update of each particle with Equation (26). The fitness value of each particle $f\left(a_{i}^{1}\right)$, which is calculated from Equation (25), is compared to the initial individual extremum $f\left(p_{i}^{0}\right)$. If $f\left(a_{i}^{1}\right)$ > $f\left(p_{i}^{0}\right)$, the individual extremum is replaced with $f(a_{i}^{1}$), or else, the individual extremum is invariant. Subsequently, the local extremum ($g_{loc}^{1}$) and the global extremum $g_{best}^{1}$ of the population at step 1 are selected with Equation (27). When the global extremum $g_{best}^{1}$ is equal to M, the algorithm breaks; or else, the iteration continues and *k = k* + 1.
Step 3. PSO algorithm termination. The iteration will not break until the iteration number k = $k_{max}$ or the current global extremum $g_{best}^{k}$ is equal to M.

**Figure 5:**
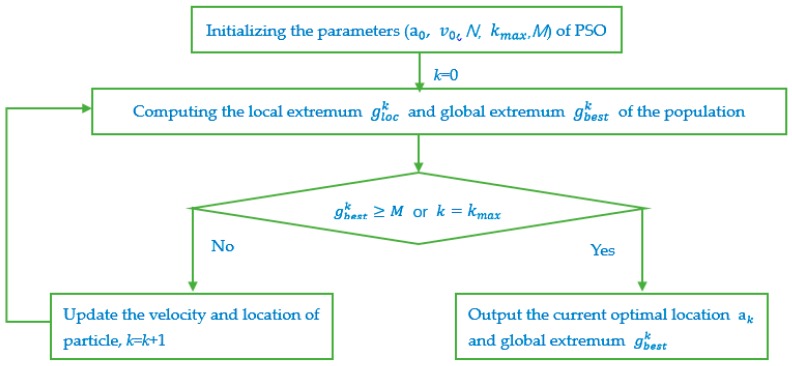
Flow chat of solving the optimal model with Particle Swarm Optimization (PSO) algorithm.

The flow chat of above optimization procedure is shown in Figure 5.

## 4. Verifications through Simulations and Experimentation

In order to assess the optimal placement effectiveness of the sensors proposed in this paper, simulations and model experimentation are performed. In Section 4.1, a finite element model of a thin-walled beam with a circular cross-section is constructed through high-fidelity direct Finite Element analyses software (ANSYS 14.5, ANSYS, Southpointe, PA, USA), used to assess the robustness of the optimal placement schemes of the strain sensors. In Section 4.2, a wing frame model is tested under different static loads and presented, in order to assess the feasibility of iFEM for sensing wing frame deformation under the condition that the sensors are optimally placed. 

### 4.1. Simulation Verification of Beam

**Figure 6:**
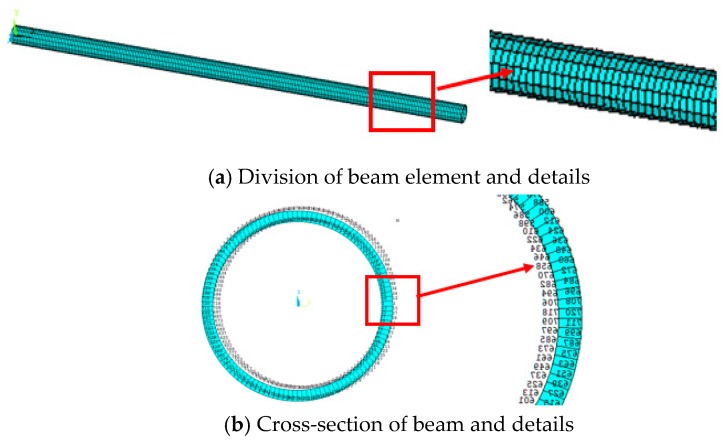
Finite element model of beam and its cross-section division.

To model the thin-walled beam structure, we made use of the Beam188 elements module with two nodes, based on the Timoshenko beam theory. The Young’s modulus is E=75,000 MPa, the Poisson ratio is v=0.3 and the density is $\rho =2557\;\mathrm{kg}/m^{3}$. The beam length is 660 mm, the external radius is Rext=13 mm and the thickness is s=1.5 mm. The finite element model of the beam is divided into 200 elements and the cross-section of the beam is divided into 120 sectors (see Figure 6).

For comparison, two normal sensors placements (C1 and C2) are quoted from the literature [28], while two optimal results (C3 and C4) are obtained from the Equation (24) model with PSO. The four placements of the strain sensors are presented in Table 1, where C1 and C3 are suited to the end-node loading, C2 and C4 are suited to the uniformly distributed loading and $f\left(A_{\left(x,\theta ,\beta \right)}\right)$ indicates the function value in the optimal model of Equation (24).

**Figure 7:**
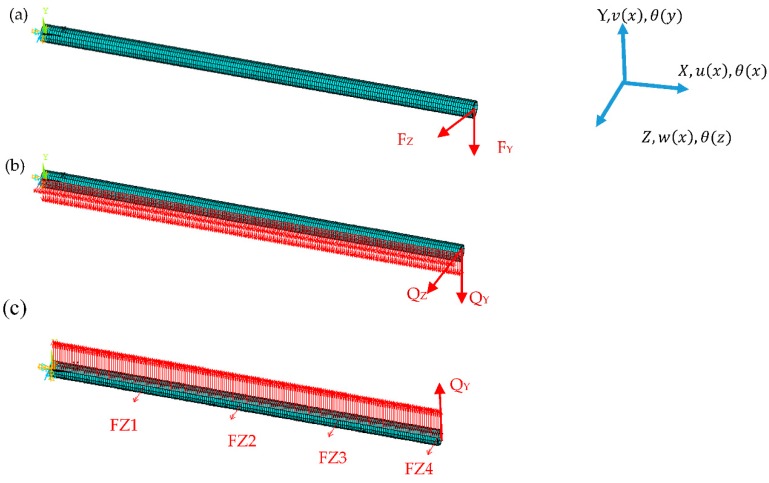
Loading cases on cantilever beam: (**a**) end-node forces: FY = −100 N, FZ = 80 N; (**b**) uniform distribution loads: Q_Z_ = 1.5 N, Q_Y_ = −1 N; (**c**) complex loads: uniform loads (Q_Y_ = 1 N) in direction Y and four equal node forces in direction Z (FZ1 = FZ2 = FZ3 = FZ4 = 150 N).

Three different static loading tests are performed on the beam model. For the first test, the two end-node forces are loaded on the model, −100 N in direction Y and 80 N in direction Z (Loading A, Figure 7a); for the second test, two uniform distribution forces are loaded along the beam: −1 N in direction Y and 1.5 N in direction Z (Loading B, Figure 7b); for the third test, complex loads (Loading C) producing uniform distribution of loads (1 N in direction Y) with four equal node forces are loaded on the beam (150 N in direction Z, Figure 7c).

In lieu of the experimentally measured surface strains and the deformation results, the corresponding data are extracted from ANSYS. The deformations calculated from iFEM, the deformations extracted from ANSYS, the absolute error (AE) and the percentage errors (PE) for the free-end displacements along with the rotations of the beam model are presented in Table 2, Table 3 and Table 4. The displacements and rotations are expressed in mm and rad, respectively.

(28) $$\;PE=\frac{\left|\delta^{iFEM}-\delta^{ANSYS}\right|}{\left|\delta^{ANSYS}\right|}\%\;AE=\left|\delta^{iFEM}-\delta^{ANSYS}\right|\;$$

where, δ=(u(x), v(x), w(x), θ(x), θ(y), θ(z)); the superscript ‘iFEM’ refers to the calculated value; and ‘ANSYS’ refers to the extracted value from ANSYS.

The comparisons of Table 2, Table 3 and Table 4 demonstrate that the two optimal placements (C3 and C4) present higher accuracy (maximum percent error is below 1%) for the iFEM beam deformation calculation compared to the schemes (C1 and C2) in the literature [28]. As discussed in section 3, strain input errors $\Delta \varepsilon \left(x_{i},\theta_{i},\beta_{i}\right)$ exist in Equation (22). It is assumed that the theoretical locations, where the strain sensors are placed, are unaltered. This means that the transformation matrix $A=T\left(x_{i},\theta_{i},\beta_{i}\right)H\left(x_{i}\right)$ is invariable but the actual locations, where the strain sensors are placed, will change. Thus, the strain inputs in Equation (22) do not correspond to the theoretical locations. The difference between the theoretical locations and the actual locations of strain sensors is set:

(29) $$\Delta x_{i}\in \left[-0.05L,0.05L\right],\Delta \theta_{i}\in \left[-9^{{}^{\circ}},9^{{}^{\circ}}\right]\;\mathrm{and}\;\Delta \beta_{i}\in [-9^{{}^{\circ}},9^{{}^{\circ}}]\;(i=1,\;2,\ldots ,m).$$

The strain measurement system errors are assumed to obey the Gaussian error distributions, which have zero mean value and three-standard deviations equal to 5% of ANSYS simulation strain value, such as $\Delta \varepsilon_{i}\in \left[-5\%\varepsilon_{i},\;5\%\varepsilon_{i}\right]$. These disturbances are added 1000 times and the highest errors are selected as the worst sensing results (presented in Table 5, Table 6 and Table 7, the displacements and the rotations are expressed in mm and rad, respectively).

The comparisons from Table 5, Table 6 and Table 7 show that the two optimal sensor placement schemes of C3 and C4 are more robust than the two quoted schemes of C1 and C2. For the schemes C1 and C2, the absolute and relative errors drastically increase; the maximum relative error is 1225.16% in direction Y and the maximum absolute error is 298.55 mm in direction Z in the case of complex loads. In contrast, for the two optimal sensor placement schemes, the maximum relative error is 103.95% in direction Y in the case of complex loads, whereas the maximum relative error is below 50% in the other two loading cases. The reason why difference increases sharply between the computed results and the extracted data is that the small changes in strain inputs cause large changes in the p1 and p2 parameter calculation through Equation (22). From Table 1, it is observed that the large differences among the matrix eigenvalues for the schemes of C3 and C4 (both exceed 10) are higher compared to the schemes of C1 and C2 (both equal to approximately 1). Consequently, the distributions of eigenvalues for C3 and C4 are better compared to the C1 and C2; and the changes of the calculated results for the C3 and C4 schemes are more stable compared to C1 and C2.

### 4.2. Experimental Verification of Wing Frame

**Figure 8:**
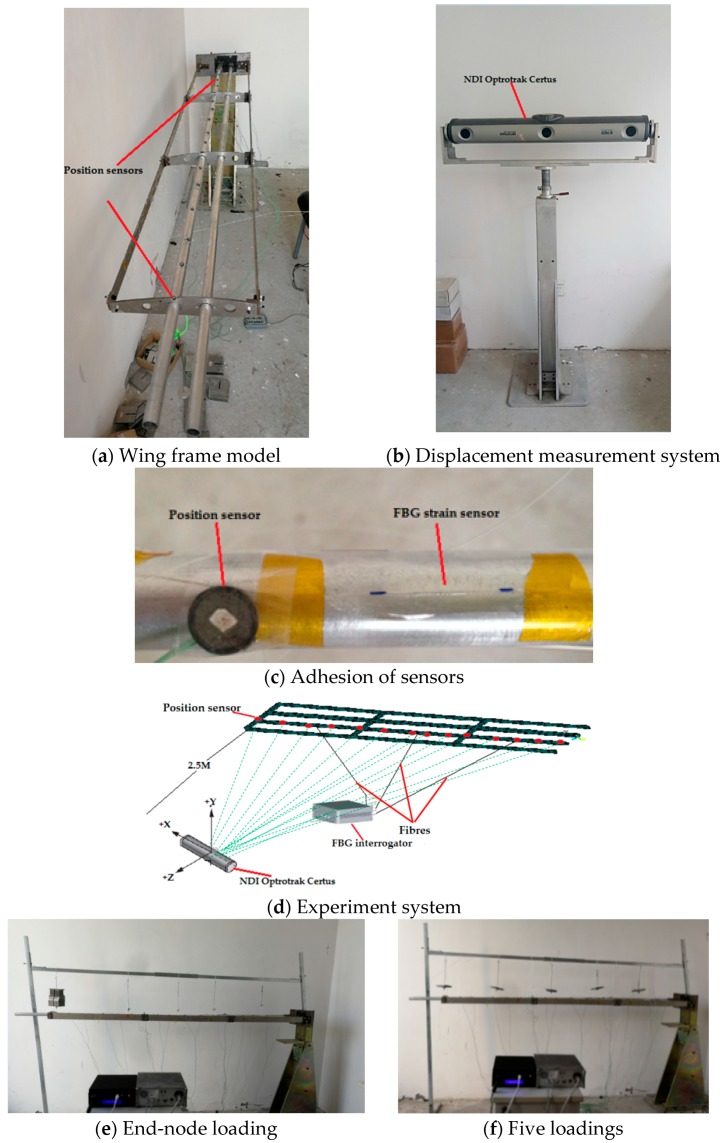
Wing frame model tests.

The experimental tests under different static loadings are performed on a wing frame model, in order to verify the feasibility and effectiveness of iFEM structural deformation sensing for two optimal placements of sensors. The frame model is made of an aluminum alloy, combining two thin-walled beams with several thin-walled plates. The length and thickness of each beam are 2 m and 1.5 mm, respectively but the external radiuses differ for the two beams: one radius is 13 mm as the main spar, whereas the other radius is 11.5 mm. The entire frame is divided into three sections and the length of each section is *L* = 666 mm (see Figure 8A). Displacement measurements are conducted at different locations along the main spar with position sensors (see Figure 8A,C), which sends the infrared lights to the CCD cameras of the 3D optical measurement instruction (see Figure 8B NDI Optrotrak Certus, NDI, Canada) to reflect the structure deformation. The accuracy of NDI Optrotrak Certus is 0.1 mm in its measurement range. The displacements captured from NDI are used to assess the accuracy of deformation sensing from the strain data with iFEM. 

The FBG strain sensor is a novel strain measurement device, based on the light wavelength shift which is caused by the FBG grating deformation generated by the tension/compressive force or change of temperature. The deformation for the unit length of the grating is labeled as the strain. Without considering the effect of the temperature change, the strain is calculated through Equation (30) [44,45].

(30) $$\varepsilon_{i}=K\times \left(\frac{\lambda_{end\left(i\right)}-\lambda_{ini\left(i\right)}}{\lambda_{ini}}\right)\;with\;K=1-P_{e}\;$$

where, $\lambda_{end\left(i\right)}$ and $\lambda_{ini\left(i\right)}$ are the wavelength shift and initial wavelength of *i-th* FBG sensor; $P_{e}$ is the photo-optical coefficient of the fiber; the strain measurement $\varepsilon_{i}$ is expressed as micro strain. 

In the model test, the experimental strain data are obtained from the strain measurement system composed of FBG strain sensors (Fiber Bragg Grating|os1100, Micron Optics, Shelburne, VT, USA) and the FBG interrogator (Optical Sensing Instrument|Si 155, Micron Optics, Atlanta, GA, USA). Twenty-four FBG strain sensors (the range of initial wavelength is [1527 nm, 1564 nm]) are placed at different locations along the main spar and used to capture the surface strain. Since the locations in placement scheme C3 are contained in scheme C4 (refer to Table 1), eight FBG sensors are placed on the surface of every section according to scheme C4. The entire experiment system is presented in Figure 8D.

For the static tests, two different loading cases are considered (see Figure 8E,F):

I. the end-node of the main spar is loaded 6 times (for the total weight of F = 5.57 KG);

II. the main spar is loaded 5 times with uniform-distributed loads (for the total weight of F = 6.02 KG).

**Figure 9:**
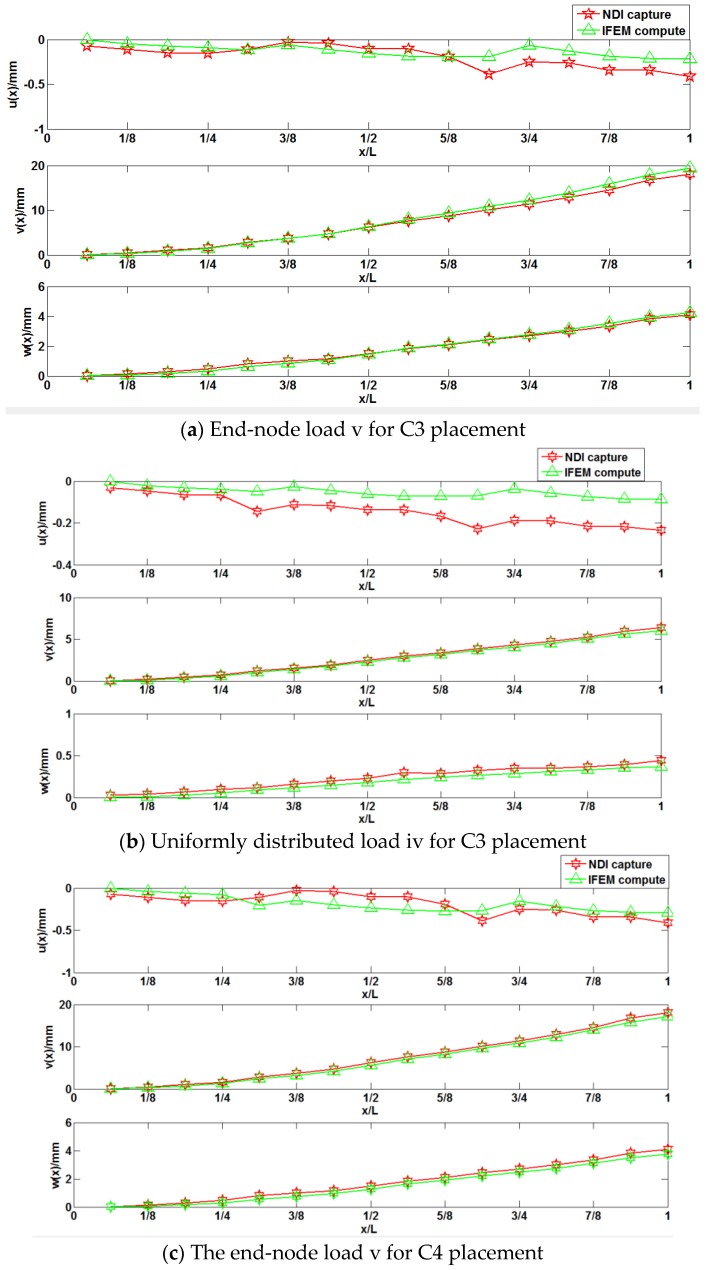

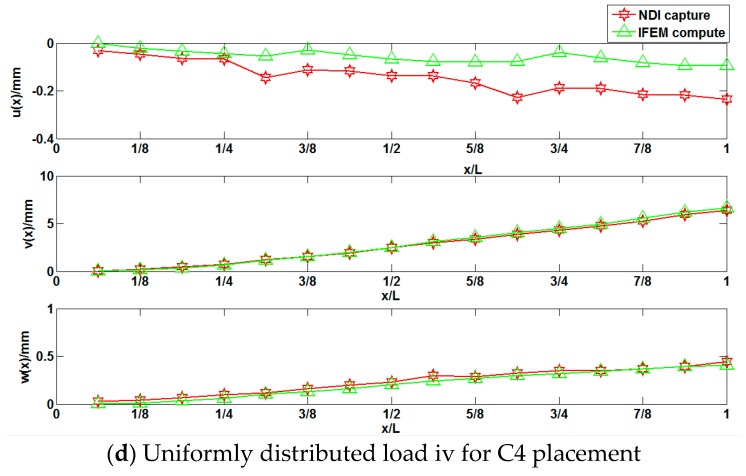
Comparisons among iFEM calculations for different placements of sensors and NDI measurements for two different loads.

The details of the above loads, the maximum deformations calculated with iFEM and the maximum deformations captured by NDI are presented in Table 8. The optical measurement instruction only captures the displacement of position sensors stuck to the structure surface. Therefore, the comparisons among iFEM calculations and NDI measurements focuses on the displacements only. To visually describe the structural deformation, the comparisons of the discrete point displacements among iFEM calculations and NDI measurements are shown in Figure 9 (for the end-node load v and the uniformly distributed load iv).

The root-mean-square difference (RMSD) is used to assess the sensing accuracy of the entire structure through iFEM [46].

(31) $$\;RMSD=\sqrt[{2}]{\overset{j}{\underset{i=1}{\sum }}{\left(disp^{NDI}\left(x_{i}\right)-disp^{iFEM}\left(x_{i}\right)\right)}^{2}/j}\;$$

where, $disp\left(x_{i}\right)$ is the displacement of one node along the beam centroidal axis in one direction; the superscript ‘NDI’ refers to the deformation values captured from NDI; ‘iFEM’ refers to the predicted values computed from in situ strain measurements with iFEM; and *j* is the number of the nodes used to describe the beam deformation. In the test, the number of nodes used to describe the beam deformation is 16. The comparisons are shown in Table 9.

The frame is divided into three sections and every section is regarded as an iFEM element, so the entire frame is calculated three times through iFEM. The calculations in the second and third sections of the frame are affected by the computation in the first section. Consequently, accumulative errors occur during the deformation sensing of the entire frame structure. In Table 8, it is observed that the maximum differences of displacements among the iFEM calculations and the NDI captures are below 12%; for the main deformation *v(x)* under all loading cases, the errors remain at approximately 5%, when the deformations increase. Although the percentage errors are relatively high, the RMSD is acceptable for the C3 and C4 schemes: (1) under the end-node loads, the maximum RMSD is 0.644 mm for the main deformation v(x) and 0.234 mm for the minor deformation w(x); (2) under the uniformly distributed loads, the RMSD is 0.32 mm for the main deformation v(x) and 0.219 mm for the minor deformation w(x) (refer to Table 9). The corresponding figures also demonstrate that the deformations calculated with iFEM for the sensor placements of C3 and C4 are quite similar to the deformations measured with the optical measurement instruction (NDI). Nevertheless, the number of measurement strains for C4 is higher compared to the C3 number. Therefore, more disturbances exist for the C4 than for the C3, which results in the sensing accuracy of C4 being slightly worse than the C3. Finally, it is discovered that the accuracies in the model experiment are worse compared to the simulation, which also demonstrate that the practical location errors of the strain sensors affect the deformation sensing of the entire structure. 

## 5. Conclusions

The study successfully validates the feasibility of iFEM used for deformation sensing of a wing frame. As aforementioned in the introduction, the locations where the strain sensors are placed affect the accuracy of iFEM for sensing. Therefore, in the paper, an optimal placement model of sensors is proposed to confirm the suitable placement of sensors for the accuracy and stability of iFEM of sensing frame deformation to be maintained. Following experimental test, the results demonstrate that the iFEM is able to precisely sense the deformation of the wing frame for the two optimal placement schemes of C3 and C4. Nevertheless, it is found that certain limits exist for the iFEM application: (1) the cross-section of the beam must be constant along the central axis of the beam and the section must be un-deformed when the loads are applied on the beam; (2) the boundary condition of the sensing equation must be known. Moreover, the test results also demonstrate that the strain measurement system errors (combining location errors of sensors with measurement errors of sensors) affect the accuracy of sensing results. Further works will be focused on the location calibrations of the strain sensors, as well as the relationship between the structural strain and the wavelength shift of the FBG sensor. 

## Figures and Tables

**Table 1:** Description of strain sensor configurations. For each configuration, axial locations and orientations of sensors reported as ($x_{i},\theta_{i},\beta_{i}$) and angles expressed in degrees.

	End-Node Loading		Uniformly Distributed Loading	
	C1	C3	C2	C4
$\varepsilon_{1}$	(0.5L,−120,0)	(0.2L,−120,0)	(0.33L,−120,45)	(0.2L,−120,0)
$\varepsilon_{2}$	(0.5L,−120,45)	(0.2L,0,0)	(0.5L,−120,0)	(0.2L,0,0)
$\varepsilon_{3}$	(0.5L,0,0)	(0.2L,120,0)	(0.5L,−120,45)	(0.2L,120,0)
$\varepsilon_{4}$	(0.5L,0,45)	(0.8L,−120,0)	(0.5L,0,0)	(0.5L,−120,0)
$\varepsilon_{5}$	(0.5L,120,0)	(0.8L,0,45)	(0.5L,0,45)	(0.5L,0,0)
$\varepsilon_{6}$	(0.5L,120,45)	(0.8L,120,0)	(0.5L,120,0)	(0.8L,−120,0)
$\varepsilon_{7}$	-	-	(0.5L,120,45)	(0.8L,0,45)
$\varepsilon_{8}$	-	-	(0.66L,120,45)	(0.8L,120,0)
$f\left(A_{\left(x,\theta ,\beta \right)}\right)$	1.07	11.45	1.09	10.53

**Table 2:** Comparisons among deformations computed with inverse Finite Element Method (iFEM) and deformations extracted from ANSYS for end-node load (Loading A).

Deformation	u(x)	v(x)	w(x)	θ(x)	θ(y)	θ(z)
ANSYS	0	−14.74	11.79	0	−0.03	−0.03
iFEM_C1_	0	−14.88	11.90	0	−0.03	−0.03
AE_C1_	0	0.14	0.11	0	0	0
PE_C1_	0%	0.95%	0.93%	0%	0%	0%
iFEM_C3_	0	−14.68	11.74	0	−0.03	−0.03
AE_C3_	0	0.06	0.05	0	0	0
PE_C3_	0%	0.41%	0.42%	0%	0%	0%
iFEM_C2_	0	−17.25	14.80	0	−0.03	−0.03
AE_C2_	0	2.51	3.01	0	0	0
PE_C2_	0%	17%	25.5%	0%	0%	0%
iFEM_C4_	0	−14.67	11.74	0	−0.03	−0.03
AE_C4_	0	0.07	0.05	0	0	0
PE_C4_	0%	0.47%	0.42%	0%	0%	0%

AE = absolute error, PE = percentage error.

**Table 3:** Comparisons among deformations computed with iFEM and deformations extracted from ANSYS for uniform distribution loads (Loading B).

Deformation	u(x)	v(x)	w(x)	θ(x)	θ(y)	θ(z)
ANSYS	0	−11.14	16.71	0	−0.03	−0.02
iFEM_C1_	0	−9.46	14.19	0	−0.02	−0.02
AE_C1_	0	1.68	2.52	0	0.01	0
PE_C1_	0%	15.08%	15.08%	0%	33.33%	0%
iFEM_C3_	0	−11.2	16.81	0	−0.03	−0.02
AE_C3_	0	0.06	0.1	0	0	0
PE_C3_	0%	0.54%	0.6%	0%	0%	0%
iFEM_C2_	0	−15.56	20.07	0	−0.04	−0.03
AE_C2_	0	4.42	3.36	0	0.01	0.01
PE_C2_	0%	39.68%	20.11%	0%	33.33%	50%
iFEM_C4_	0	−11.05	16.58	0	−0.03	−0.02
AE_C4_	0	0.09	0.13	0	0	0
PE_C4_	0%	0.81%	0.78%	0%	0%	0%

AE = absolute error, PE = percentage error.

**Table 4:** Comparisons among deformations computed with iFEM and deformations extracted from ANSYS for complex loads (Loading C).

Deformation	u(x)	v(x)	w(x)	θ(x)	θ(y)	θ(z)
ANSYS	0	11.13	44.35	0	−0.09	0.02
iFEM_C1_	0	9.46	36.04	0	−0.07	0.02
AE_C1_	0	1.67	8.31	0	0.02	0
PE_C1_	0%	15%	18.74%	0%	22.22%	0%
iFEM_C3_	0	11.21	44.41	0	−0.09	0.02
AE_C3_	0	0.08	0.06	0	0	0
PE_C3_	0%	0.72%	0.14%	0%	0%	0%
iFEM_C2_	0	10.77	45.13	0	−0.1	0.02
AE_C2_	0	0.36	0.78	0	0.01	0
PE_C2_	0%	3.23%	1.76%	0%	11.11%	0%
iFEM_C4_	0	11.05	43.91	0	−0.09	0.02
AE_C4_	0	0.08	0.44	0	0	0
PE_C4_	0%	0.72%	0.99%	0%	0%	0%

AE = absolute error, PE = percentage error.

**Table 5:** Comparisons among deformations computed with iFEM and deformations extracted from ANSYS in case of end-node loads (Loading A). Strain inputs contained errors.

Deformation	u(x)	v(x)	w(x)	θ(x)	θ(y)	θ(z)
ANSYS	0	−14.74	11.79	0	−0.03	−0.03
iFEM_C1_	0.04	6.7	50.37	0	−0.03	−0.03
AE_C1_	0.04	21.44	38.58	0	0	0
PE_C1_	-	145.45%	327.23%	0%	0%	0%
iFEM_C3_	0	−17.61	7.91	0	−0.02	−0.04
AE_C3_	0	2.87	3.88	0	0.01	0.01
PE_C3_	0%	19.47%	32.91%	0%	33.33%	33.33%
iFEM_C2_	0.04	−113.7	154.58	0	−0.38	−0.25
AE_C2_	0.04	98.96	142.79	0	0.35	0.22
PE_C2_	-	671.37%	1211.11%	0%	1166.67%	733.33%
iFEM_C4_	0.11	−16.7	12.85	0	−0.02	−0.04
AE_C4_	0.11	1.96	1.06	0	0.01	0.01
PE_C4_	-	13.30%	8.99%	0%	33.33%	33.33%

AE = absolute error, PE = percentage error.

**Table 6:** Comparisons among deformations computed with iFEM and deformations extracted from ANSYS in case of uniform distribution loads (Loading B). Strain inputs contained errors.

Deformation	u(x)	v(x)	w(x)	θ(x)	θ(y)	θ(z)
ANSYS	0	−11.1	16.6	0	−0.03	−0.02
iFEM_C1_	0	5.89	40.25	0	−0.03	−0.01
AE_C1_	0	16.99	23.65	0	0	0.01
PE_C1_	0%	153.06%	142.47%	0%	0%	50%
iFEM_C3_	0.05	−15.64	13.56	0	−0.03	−0.03
AE_C3_	0.05	4.54	3.04	0	0	0.01
PE_C3_	-	40.9%	18.31%	0%	0%	50%
iFEM_C2_	0	−93.11	118.45	0	−0.29	−0.20
AE_C2_	0	82.01	101.85	0	0.26	0.18
PE_C2_	0%	738.83%	613.55%	0%	866.67%	900%
iFEM_C4_	0.16	−13.48	17.58	0	−0.03	−0.03
AE_C4_	0.16	2.38	0.98	0	0	0.01
PE_C4_	-	21.44%	5.9%	0%	0%	50%

AE = absolute error, PE = percentage error.

**Table 7:** Comparisons among deformations computed with iFEM and deformations extracted from ANSYS in case of complex loads (Loading C). Strain inputs contained errors.

Deformation	u(x)	v(x)	w(x)	θ(x)	θ(y)	θ(z)
ANSYS	0.0	11.13	44.35	0	−0.09	0.02
iFEM_C1_	0.06	54.35	−12.94	0	−0.06	0.01
AE_C1_	0.06	43.22	57.29	0	0.03	0.01
PE_C1_	-	388.32%	129.18%	0%	33.33%	50%
iFEM_C3_	0.26	1.02	36.47	−0.05	−0.08	−0.01
AE_C3_	0.26	10.11	7.88	0.05	0.01	0.03
PE_C3_	-	90.84%	17.77%	-	11.11%	150%
iFEM_C2_	0	−125.23	342.9	0	−0.87	−0.23
AE_C2_	0	136.36	298.55	0	0.78	0.25
PE_C2_	0%	1225.16%	673.17%	0%	866.67%	1250%
iFEM_C4_	0.04	−0.44	40.95	0	−0.09	0
AE_C4_	0.04	11.57	3.4	0	0	0.02
PE_C4_	-	103.95%	7.67%	0%	0%	100%

AE = absolute error, PE = percentage error.

**Table 8:** Loads for static tests (loads expressed in kg and displacements expressed in mm).

	End-Node Loads						Uniformly Distributed Loads				
Times	i	ii	iii	iv	v	vi	i	ii	iii	iv	v
loads	1.53	2.22	3.11	4.05	4.98	5.57	1.19	2.66	3.64	4.62	6.02
$u{\left(x\right)}_{NDI}$	−0.1	−0.2	−0.3	−0.3	−0.4	−0.4	−0.1	−0.1	−0.1	−0.2	−0.2
$u{\left(x\right)}_{iFEM_{C3}}$	0.0	−0.1	−0.1	−0.2	−0.2	−0.3	0	0	−0.1	−0.1	−0.1
$u{\left(x\right)}_{\mathrm{PEC}3}$	100%	50%	66.6%	33.3%	50%	25%	100%	100%	0%	50%	50%
$u{\left(x\right)}_{iFEM_{C4}}$	0.0	0.0	−0.1	−0.2	−0.3	−0.3	0	0	0	−0.1	−0.1
$u{\left(x\right)}_{\mathrm{PEC}4}$	100%	100%	66.6%	33.3%	25%	25%	100%	100%	100%	50%	50%
$v{\left(x\right)}_{NDI}$	3.8	6.1	9.5	13.2	16.1	18.1	0.9	2.5	3.9	5.0	6.4
$v{\left(x\right)}_{iFEM_{C3}}$	3.7	5.7	9.2	13.9	17.4	19.2	1.0	2.7	4.1	5.2	6.7
$v{\left(x\right)}_{\mathrm{PEC}3}$	2.6%	6.6%	3.2%	5.3%	4.3%	5.0%	11.1%	8%	5.1%	4%	4.7%
$v{\left(x\right)}_{iFEM_{C4}}$	4.0	6.4	9.9	12.6	15.3	17.1	0.8	2.2	3.6	4.7	6.0
$v{\left(x\right)}_{\mathrm{PEC}4}$	5.3%	4.9%	4.2%	4.5%	4.9%	5.5%	11.1%	12%	7.7%	6%	6.3%
$w{\left(x\right)}_{NDI}$	0.8	1.3	2.1	2.9	3.7	4.1	−0.1	0.1	0.2	0.3	0.4
$w{\left(x\right)}_{iFEM_{C3}}$	0.9	1.3	2.2	3.1	3.9	4.3	0	0.1	0.2	0.2	0.4
$w{\left(x\right)}_{\mathrm{PEC}3}$	11.3%	0%	4.8%	6.9%	5.4%	4.9%	100%	0%	0%	33.3%	0%
$w{\left(x\right)}_{iFEM_{C4}}$	0.7	1.2	2.0	2.7	3.4	3.8	0	0.1	0.1	0.3	0.4
$w{\left(x\right)}_{\mathrm{PEC}4}$	11.3%	7.7%	4.8%	6.9%	8.1%	7.3%	100%	0%	50%	0%	0%

**Table 9:** Root-mean-square difference (RMSD) of iFEM calculation for different loads (RMSD expressed in mm).

	End-Node Loads						Uniformly Distributed Loads				
Times	i	ii	iii	iv	v	vi	i	ii	iii	iv	v
$u{\left(x\right)}_{NDI}$	−0.1	−0.2	−0.3	−0.3	−0.4	−0.4	−0.1	−0.1	−0.1	−0.2	−0.2
$RMSD_{iFEM_{C3}}$ for *u(x)*	0.068	0.097	0.151	0.205	0.277	0.296	0.018	0.037	0.062	0.079	0.102
$RMSD_{iFEM_{C4}}$ for *u(x)*	0.069	0.098	0.152	0.207	0.280	0.298	0.018	0.038	0.062	0.080	0.103
$v{\left(x\right)}_{NDI}$	3.8	6.1	9.5	13.2	16.1	18.1	0.9	2.5	3.9	5.0	6.4
$RMSD_{iFEM_{C3}}$ for *v(x)*	0.166	0.176	0.292	0.518	0.381	0.416	0.132	0.169	0.230	0.261	0.308
$RMSD_{iFEM_{C4}}$ for *v(x)*	0.196	0.246	0.357	0.541	0.641	0.644	0.134	0.174	0.236	0.269	0.320
$w{\left(x\right)}_{NDI}$	0.8	1.3	2.1	2.9	3.7	4.1	−0.1	0.1	0.2	0.3	0.4
$RMSD_{iFEM_{C3}}$ for *w(x)*	0.049	0.055	0.056	0.169	0.142	0.115	0.061	0.131	0.107	0.119	0.155
$RMSD_{iFEM_{C4}}$ for *w(x)*	0.09	0.09	0.164	0.317	0.208	0.234	0.070	0.156	0.146	0.169	0.219
